# Construction Notes

**Published:** 1893-11-04

**Authors:** 


### CONSTRUCTION NOTES.

**Extension at the North Infirmary, Cork.**

A new wing has just been added to the infirmary, which is built as a consequence to a bequest of £28,000 made some three years ago by the late Lady Combermere, a daughter of Dr. Gibbings, one of the medical officers of the institution, in the early part of the century. The new wing is a handsome addition to the building already existing. It is divided into two wards, the Gibbings Ward being for male patients and the Combermere Ward for females. In each ward are eighteen beds The extra accommodation specially for accident patients was very badly needed, but, as only a small portion of the bequest could be used for the endowment of the two wards, their maintenance will mean an additional burthen on the General Management Committee, who, to a great extent, depend on voluntary aid. The architect for this extension was Mr. Arthur Hill, B.E.C.E, of Cork, the contractors, also local, being Messrs. E. and P. Plynn.

